# Joint Infrared Target Recognition and Segmentation Using a Shape Manifold-Aware Level Set

**DOI:** 10.3390/s150510118

**Published:** 2015-04-29

**Authors:** Liangjiang Yu, Guoliang Fan, Jiulu Gong, Joseph P. Havlicek

**Affiliations:** 1 School of Electrical and Computer Engineering, Oklahoma State University, Stillwater, OK 74078, USA; E-Mail: liangjiang.yu@okstate.edu; 2 School of Mechatronical Engineering, Beijing Institute of Technology, Beijing 100081, China; E-Mail: lujiugong@bit.edu.cn; 3 School of Electrical and Computer Engineering, University of Oklahoma, Norman, OK 73019, USA; E-Mail: joebob@ou.edu

**Keywords:** infrared ATR, level set, shape modeling, particle swarm optimization

## Abstract

We propose new techniques for joint recognition, segmentation and pose estimation of infrared (IR) targets. The problem is formulated in a probabilistic level set framework where a shape constrained generative model is used to provide a multi-class and multi-view shape prior and where the shape model involves a couplet of view and identity manifolds (CVIM). A level set energy function is then iteratively optimized under the shape constraints provided by the CVIM. Since both the view and identity variables are expressed explicitly in the objective function, this approach naturally accomplishes recognition, segmentation and pose estimation as joint products of the optimization process. For realistic target chips, we solve the resulting multi-modal optimization problem by adopting a particle swarm optimization (PSO) algorithm and then improve the computational efficiency by implementing a gradient-boosted PSO (GB-PSO). Evaluation was performed using the Military Sensing Information Analysis Center (SENSIAC) ATR database, and experimental results show that both of the PSO algorithms reduce the cost of shape matching during CVIM-based shape inference. Particularly, GB-PSO outperforms other recent ATR algorithms, which require intensive shape matching, either explicitly (with pre-segmentation) or implicitly (without pre-segmentation).

## 1. Introduction

We consider automatic target recognition (ATR) systems that detect and recognize extended targets by processing a sequence of images acquired from a passive imaging infrared (IR) sensor [1,2]. Our interest is primarily in sensors operating in the traditional 3–5 μm mid-wave IR (MWIR) or 8–12 μm long-wave IR (LWIR) bands, although our results are also applicable to those operating in the near, short-wave or far-IR bands, as well. The main functions typically performed by practical IR ATR systems of these types include detection, segmentation, feature extraction, tracking and recognition [3,4]. While these functions have historically been implemented sequentially, there is a growing recent interest in performing them jointly, so that tracking and recognition are not delayed by ambiguities in the detection process and so that inferences made by the track processor can be leveraged for both recognition and detection.

**Figure 1:**
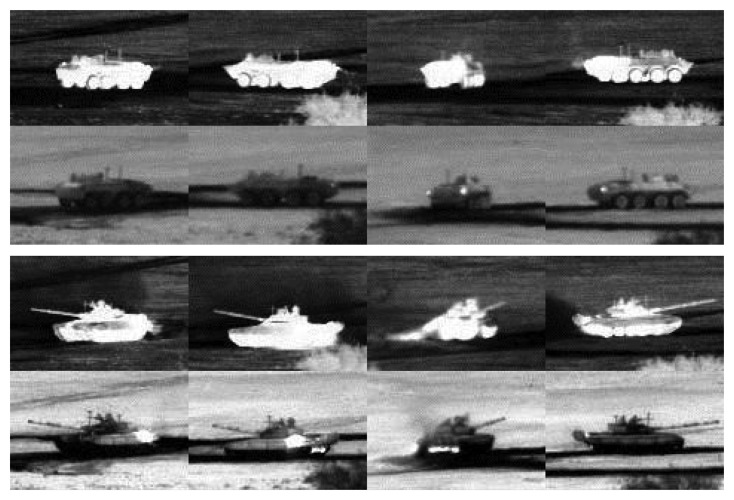
Examples of target signature variability from the Military Sensing Information Analysis Center (SENSIAC) ATR database [5]. The first and second rows show diurnal and nocturnal mid-wave IR (MWIR) images of a BTR70 personnel carrier, respectively. The third and fourth rows are diurnal and nocturnal images of a T72 main battle tank. Targets in each column are under the same view.

The infrared ATR problem presents significant challenges. Growth and processing techniques for IR detector materials, such as HgCdTe and InSb, are less mature than those for silicon, and hence, imaging IR sensors are typically characterized by higher noise and poor uniformity compared to their visible wavelength counterparts. The imagery acquired under practical field conditions often exhibits strong, structured clutter, poor target-to-clutter ratios and poor SNR. In important surveillance, security and military applications, the targets of interest may be non-cooperative, employing camouflage, decoys, countermeasures and complex maneuvers in an effort to evade detection and tracking. These difficulties are often exacerbated by the strong ego-motion of the sensor platform relative to the target. Depending on the operational waveband of the sensor, environmental conditions, such as smoke, haze, fog and rain, can result in degraded target signatures, as well as partial or full occlusions. All of these factors contribute to substantial appearance variability of the target thermal signature observed by the sensor, thereby limiting the effectiveness of approaches based on, e.g., stored libraries of static *a priori* signatures. A few examples of MWIR signature variability from the Military Sensing Information Analysis Center (SENSIAC) ATR Algorithm Development Image Database [5] are shown in Figure 1. Moreover, one would ideally like the ATR system to be capable of generalizing on the fly, so that both unknown target types and previously unseen views of known target types can be detected, tracked and recognized, at least to within an appropriate target class.

A very large number of ATR algorithms have been proposed in recent decades [3,4,6,7]. Some have been based primarily on the computation of certain types of features, such as PCA [8], edge and corner descriptors [9], wavelets [10] or deformable templates [11], while others have been driven more by a particular classification scheme, e.g., neural networks [12], support vector machines (SVM) [13] or sparse representations [14]. In the closely-related fields of computer vision and visual tracking, there have been significant developments in object detection and recognition based on visual features, including the histogram of oriented gradients (HOG) [15,16], the scale-invariant feature transform (SIFT) [17], spin images [18], patch features [19], shape contexts [20], optical flow [21] and local binary patterns [22]. Several feature point descriptors for long-wave IR data applications were evaluated in [23], including SIFT, speeded up robust features (SURF), binary robust invariant scalable keypoints (BRISK), binary robust independent elementary features (BRIEF), fast retina keypoint (FREAK), oriented features from accelerated segment test (FAST) and rotated BRIEF (ORB) features. Certain geometric, topological and spectral descriptors have been widely used, as well [24,25]. Active contour methods [26,27] and level set algorithms have also been widely used in shape-based segmentation algorithms [28–30]. Shape priors were incorporated into both active contours and level set methods to handle cases of complicated background/foreground structure or objects in [31,32]. In [33], a couplet of identity and view manifolds (CVIM) was proposed for shape modeling by generalizing nonlinear tensor decomposition in [34]. CVIM explicitly defines view and identity variables in a compact latent space and was used with particle filtering for IR tracking and recognition in [33]. Gaussian process latent variable models (GPLVMs) were also used to learn a shape prior in order to accomplish joint tracking and segmentation in [35,36], and GPLVM was further extended for IR ATR application in [37].

In this paper, we propose a new shape-constrained level set algorithm that incorporates the parametric CVIM model in a probabilistic framework for integrated target recognition, segmentation and pose estimation. The objective energy function of the level set is defined by associating CVIM with observations via a graphical model. To cope with the multi-modal property of CVIM for implicit shape matching, we first develop a particle swarm optimization (PSO) strategy [38] to optimize the energy function with respect to CVIM parameters, and then, we further propose a gradient-boosted PSO (GB-PSO) to improve the computational efficiency by taking advantage of the analytical nature of the objective function. There are two main contributions. The first one is a unified probabilistic level set framework that integrates CVIM-based implicit shape modeling and naturally supports multiple ATR tasks in one computational flow. The second one is an efficient GB-PSO algorithm that combines both gradient-based and sampling-based optimization schemes for CVIM-based implicit shape matching. Experimental results on the SENSIAC ATR database demonstrate the performance and computational advantages of the proposed GB-PSO over other CVIM-based implementations, as well as recent ATR algorithms.

The remainder of the paper is organized as follows. Related ATR methods are reviewed in Section 2. Section 3 presents the proposed recognition and segmentation framework, including the analytical formulation and development of the two PSO-based algorithms. The experimental setup and empirical performance comparison are described in Section 4, while conclusions appear in Section 5.

## 2. Related Work

**Figure 2:**
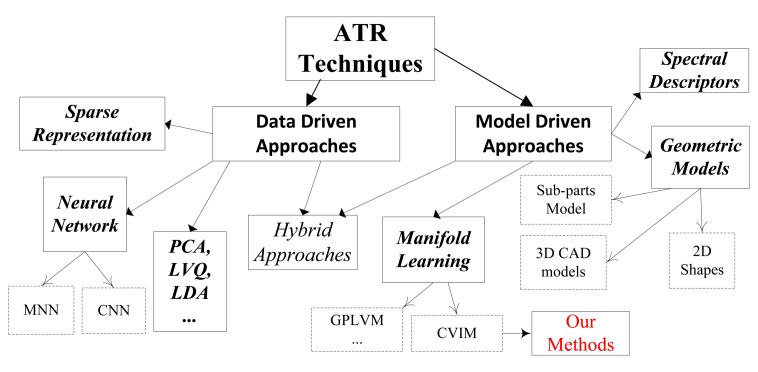
A taxonomy of ATR methods. MNN (modular neural network); CNN (convolutional neural network); LVQ (learning vector quantization); GPLVM (Gaussian process latent variable model); CVIM (couplet of view and identity manifolds).

We first categorize several recent ATR algorithms into two main groups as shown in Figure 2 and then present the motivation and contributions of our present work relative to the existing methods.

### 2.1. Data-Driven Approaches

Data-driven approaches are typically based on learning from a set of labeled real-world training data. Neural networks (NN) [39–43] are an important exemplar. With the NN-based approach, the images acquired from the sensor are treated as points in a high-dimensional vector space. The objective is then to train a large multi-layer perceptron to perform the required mapping from this space to the space of labeled training images [39]. Convolutional neural networks (CNNs) generalize the basic idea by incorporating local receptive fields, weight sharing and spatial sub-sampling to accommodate some degree of translation and local deformation [40]. Modular neural networks (MNNs) are another important generalization where a collection of several independently trained networks each make a classification decision based on local features extracted from a specific region of the image [41]. These individual decisions are then combined to arrive at the overall final ATR classification decision. Another data-driven approach is the vector quantization (VQ)-based method developed in [42,43], where each target class is trained by the learning vector quantization (LVQ) algorithm and multi-layer perceptrons (MLPs) are used for recognition. A related architecture combining several individual ATR classifiers was proposed in [12]. A K-nearest-neighbor (KNN) data-driven approach for animal recognition using IR sensors was proposed in [44]. Recently, sparse representation-based classification (SRC) methods have shown great promise in face recognition [45,46] and have also been applied to IR data for target detection [47], tracking and recognition [14,48]. The main drawbacks of these data-driven approaches are that they require large sets of training data, especially in the IR ATR applications considered here, and that the profound appearance variability of the observed target thermal signatures expected under practical field conditions tends to make dictionary selection extremely difficult.

### 2.2. Model-Driven Approaches

The model-driven approaches are based on computer-generated models (e.g., CAD models) with or without real-world sensor data for model learning. CAD models have been widely used for object segmentation, tracking and recognition [49–51]. Modern model-based ATR approaches generate target hypotheses and match the observed sensor data to the hypothesized signatures or appearance models [3]. The main idea is that a better interpretation of the scene and target can be achieved by applying intelligent reasoning while preserving as much target information as possible [52]. For example, in [53], radar features were extracted from the sensor data and used to construct a 3D model of the observed target that was compared with known models of the objects of interest to find the best match. There are also hybrid techniques [54–56] that combine both CAD models and data-driven 2D image features for model learning and inferencing. Indeed, 2D image features play an important role in many IR ATR algorithms [57], and a variety of such shape features were evaluated in [58]. One example of a hybrid technique is the multi-view morphing algorithm that was used in [59] to construct a view morphing database in an implicit way.

A number of manifold learning methods have also been proposed for shape modeling and have been applied recently in object tracking and recognition [60]. Elliptic Fourier descriptors were used in [35] to model shapes as sums of elliptic harmonics, and the latent space of target shapes was learned through GPLVMs. In [61], a level set framework was developed to optimize a pixel-wise posterior in the shape latent space in order to achieve simultaneous segmentation and tracking. A similarity space was added in [36] to handle multi-modal problems where an efficient discrete cosine transform (DCT)-based shape descriptor was used for manifold learning. A shape model called the couplet of view and identity manifolds (CVIM) that represents the target view and identity variables on a coupled pair of manifolds was proposed in [33] for joint target tracking and recognition in IR imagery. In [37], a probabilistic level set framework with shape modeling was proposed for target tracking and recognition, where a motion model was used in a particle filter-based sequential inference process. Sampling was performed in a local area predicted by the motion model, thereby alleviating the multi-modal optimization problem.

### 2.3. Research Motivation

Motivated by [33,36,37], our focus here is on developing a model-driven approach by combining relatively simple CAD models with advanced manifold learning for robust ATR to reliably segment and recognize target chips in the sequence of images acquired from an imaging IR sensor. More specifically, our goal is to incorporate CVIM into a probabilistic level set framework with shape-constrained latent space to achieve joint and seamless target segmentation, recognition and pose estimation with an analytical formulation that facilitates efficient sampling-based or gradient-based global solution of the multi-modal optimization problem. This leads to a new approach that does not require labeled training data and is free from the need of any explicit feature extraction technique. Unlike many ATR algorithms, including [33], it is also free from the need for auxiliary background rejection or pre-segmentation processing; with our proposed methods, target segmentation instead becomes a useful byproduct of the joint ATR inference process.

## 3. Proposed Methods

In this section, we first introduce a probabilistic formulation of the proposed shape-constrained level set framework. This is followed by a brief review of CVIM for shape modeling. We then propose two PSO algorithms for joint ATR optimization. The first is a standard PSO that involves CVIM-based sampling for shape interpolation, while the second is a gradient-boosted PSO (GB-PSO) that implements a gradient-based search in the CVIM latent space.

### 3.1. Problem Formulation

**Figure 3:**
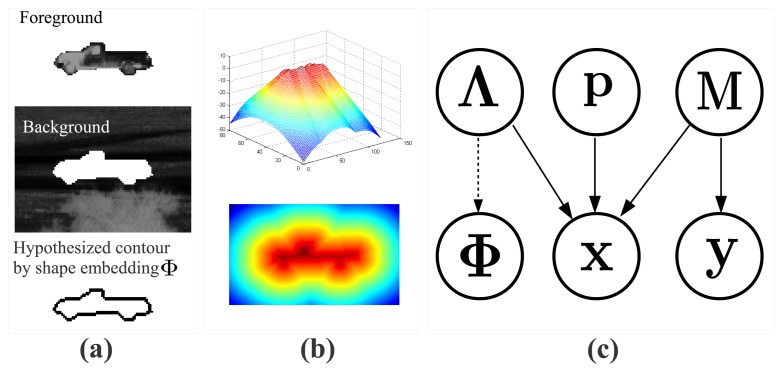
(**a**) Representation of an IR target by a hypothesized shape contour that separates the foreground and background regions; (**b**) shape embedding function **Φ** represented by the signed distance transform. **Φ** is generated by CVIM given the parameter vector **Λ**, which contains the view angles **Θ** and identity variable *α*; (**c**) The proposed probabilistic level set framework, where **p** is the target centroid in image coordinates, *M* is the foreground/background model, **x** is a pixel location in image coordinates and **y** is a pixel intensity value. The dashed line represents the CVIM-based mapping from the latent shape space **Λ** to the shape embedding function **Φ**.

Implicit contour and level set methods have been proven effective for image segmentation by optimizing an energy function, which represents the contour of an object appearing in the scene. A common approach is to compute the segmentation by optimizing the shape embedding function **Φ** [62]. The basic idea is to initialize a shape contour and then minimize the energy function related to **Φ** along the gradient direction. A probabilistic level set segmentation framework was proposed in [61], where, as illustrated in Figure 3a, an energy function called the pixel-wise posterior was defined to represent the image as a bag of pixels with the background and foreground models obtained from **Φ** [63].

Here, we extend the model from [61] to obtain a new shape-constrained level set segmentation method by incorporating the CVIM shape model parameterized by **Λ** = [**Θ***^T^*, *α*]*^T^*, which explicitly represents the target identity variable *α* and azimuth/elevation view angles **Θ** = [θ, ϕ]*^T^*, thus inherently supporting joint target recognition, segmentation and pose estimation. We derive a new joint probability density function:

(1) P(x,y,Λ,p,M)=P(x|Λ,p,M)P(y|M)P(M)P(Λ)P(p)

where *M* is the foreground/background model, **p** is the location of the target centroid in image coordinates, *P*(**p**) is the prior probability of the target centroid location, which is assumed uniform. **x** is a pixel location in image coordinates and **y** is the pixel intensity. The intensity is usually scalar-valued for the case of an imaging MWIR or LWIR sensor, but may generally be vector-valued in our framework and formulation.

By marginalizing over the foreground/background model *M* [61] and using the logarithmic opinion pool [64], we formulate a new pixel-wise posterior:

(2) $$P\left(\Lambda ,\text{p}|\Omega \right)=\prod_{i=1}^{N}\left\{P\left({\text{x}}_{i}|\Lambda ,\text{p},{\text{y}}_{i}\right)\right\}\cdot P\left(\Lambda \right)P\left(\text{p}\right)$$

where Ω = {**x**, **y**} is a small IR chip cropped from the IR frame acquired by the sensor, *N* is the number of pixels in the chip and *i* is the pixel index. Because we are focused on small IR chips that contain a target, we localize the target centroid **p** after segmentation and recognition. Therefore, **p** is omitted in the following. As in [61], *P*(**x***_i_*|**Λ**, **y***_i_*) in Equation (2) may be expressed according to:

(3) $$P\left({\text{x}}_{i}|\Lambda ,{\text{y}}_{i}\right)=H_{\epsilon }\left(\Phi_{{\text{x}}_{i}}\right)P_{f}+\left(1-H_{\epsilon }\left(\Phi_{{\text{x}}_{i}}\right)\right)P_{b}$$

where **Φ** is the shape embedding function generated from CVIM given **Λ** (in the form of a signed distance function, as shown in Figure 3b), *H*_ϵ_(·) is the smoothed Heaviside step function and **Φ**_**x***i*_ is the value of **Φ** at pixel location **x***_i_*. In Equation (3),

(4) $$P_{f}=\frac{P\left({\text{y}}_{i}|M_{f}\right)}{\eta_{f}P\left(y_{i}|M_{f}\right)+\eta_{b}P\left(y_{i}|M_{b}\right)}$$

and

(5) $$P_{b}=\frac{P\left(y_{i}|M_{b}\right)}{\eta_{f}P\left(y_{i}|M_{f}\right)+\eta_{b}P\left(y_{i}|M_{b}\right)}$$

where η*_f_* and η*_b_* are the number of pixels belonging to the foreground and background regions respectively and where *P*(**y**|*M_f_*) and *P*(**y**|*M_b_*) are the foreground and background appearance models, which are represented by histograms.

The goal of the shape-constrained level set optimization is then to maximize Equation (2) with respect to **Λ** according to:

(6) $$\Lambda *=\arg\underset{\Lambda }{\max}P\left(\Lambda |\Omega \right)$$

The calculus of variations could be applied to compute the derivative of Equation (2) with respect to **Λ**. However, due to the multi-modal nature of the CVIM-based shape modeling, we develop a PSO-based optimization framework to search for the optimal latent variable Λ* that maximizes Equation (2). To further enhance the efficiency, we then develop a gradient-boosted PSO (GB-PSO) method that provides faster optimization by taking advantage of the parametric nature of CVIM.

### 3.2. DCT-Enhanced CVIM Shape Modeling

**Figure 4:**
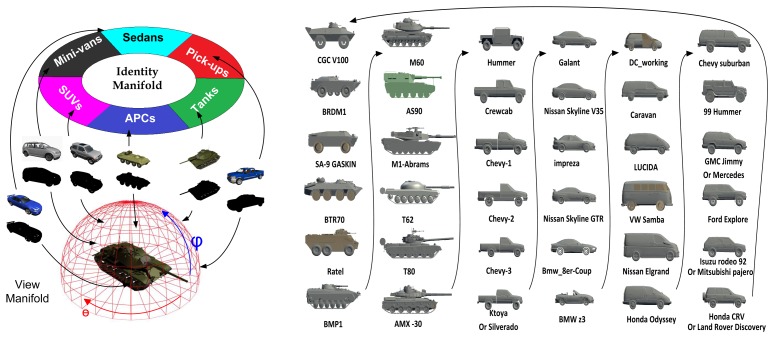
CVIM learning. (**Left**) The couplet of view and identity manifold (CVIM) shape generative model [33]; (**Right**) six CVIM CAD training models for each of six target classes.

In this section, we briefly review the CVIM [33] and then extend it to accommodate DCT-based shape descriptors for learning and inference. The CVIM can be learned from a set of 2D shape silhouettes [65] created by a series of 3D CAD models by a nonlinear kernelized tensor decomposition, as shown in Figure 4. Here, we use six models for each of six target classes. The CVIM consists of a hemisphere-shaped view manifold and a closed-loop identity manifold in the tensor coefficient space. Two practical considerations lead to this heuristic simplification of the identity manifold. First, the SENSIAC targets of interest in this work are all man-made vehicles that exhibit distinct inter-class appearance similarities. Second, these similarities can be leveraged to judiciously order the classes along a 1D closed loop manifold in order to support convenient identity inference, as shown in Figure 4. In [33], a class-constrained shortest-closed-path method was proposed to deduce an optimal topology ensuring that targets of the same class or of similar shapes remain close along the identity manifold (*i.e.*, armored personnel carriers (APCs) → tanks → pick-ups → sedans → minivans → SUVs → APCs).

The original CVIM is learned as follows: (1) given a set of silhouettes (represented by the signed distance transform) from *N_m_* target types under *N_c_* views, a mapping from a conceptual hemispherical view manifold **Θ** to the high dimensional data is learned using radial basis functions (RBFs) Ψ(**Θ**) for each target shape. (2) by stacking the collection of these mappings for all target shapes and applying the high-order singular value decomposition (HOSVD), we obtain a core tensor **A** and *N_m_* identity vectors for all training types in the tensor coefficient space **i***_m_* (*m* = 1, 2,…, *N_m_*); (3) a mapping from the coefficient vector space to a 1D closed loop identity manifold *α* is then constructed using the optimal identity manifold topology, where each training target type **i***_m_* is represented by an identity vector **i**(*α_m_*) associated with a point along the identity manifold. For any arbitrary *α* ∈ [0, 2*π*), we can then obtain a corresponding identity vector **i**(*α*) from the two closest training identity vectors **i**(*α_m_*) and **i**(*α_m_*_+1_) by applying cubic spline interpolation along the identity manifold. The CVIM model was tested against the SENSIAC database [5] for target tracking and recognition in [33], where the experimental results validated its efficacy both qualitatively and quantitatively.

**Figure 5:**
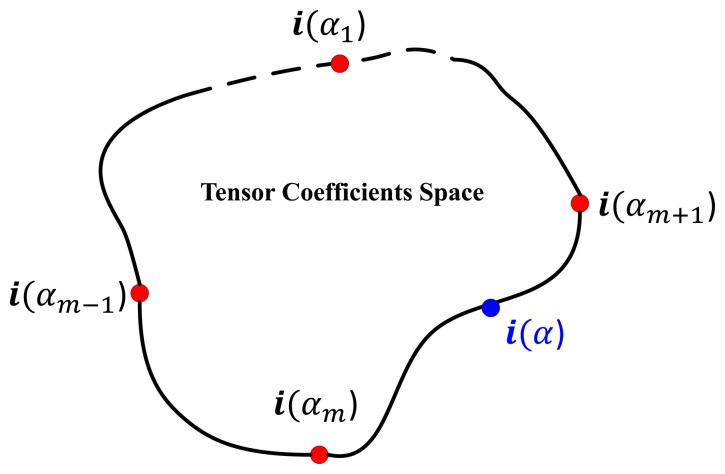
Cubic spline interpolation along the identity manifold in CVIM.

Here, we reduce the inference complexity of the original CVIM method [33] by replacing the silhouettes used for training with the simple, but efficient DCT-based shape descriptor proposed in [36]. Thus, each training shape (in the form of the signed distance transform) is represented by a small set of 2D DCT coefficients reshaped into a column vector, where, e.g., only the top 10% of the DCT coefficients that are largest in magnitude are retained. The CVIM can then be learned using the same process as before to represent the sparse DCT coefficient vectors 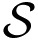
*_DCT_* of the training targets by **Λ** = [**Θ***^T^*, *α*]*^T^* according to:

(7) $$S_{\text{DCT}}\left(\Lambda \right)=S_{\text{DCT}}\left(\alpha ,\Theta \right)=\text{A}\times_{3}\text{i}\left(\alpha \right)\times_{2}\Psi \left(\Theta \right)$$

where **A** is a core tensor obtained by tensor decomposition, ×*_n_* is the mode-*n* tensor multiplication, α and **Θ** = [θ, ϕ]*^T^* are the identity and view latent variables on the identity and view manifolds, respectively, and θ and ϕ are the azimuth and elevation angles. For an arbitrary α, the associated 1 × *N_m_* identity (row) vector **i**(α) in Equation (7) can be interpolated as:

(8) $$\begin{array}{ll} \text{i}\left(\alpha \right)={\text{a}}_{m}{\left(\alpha -\alpha_{m}\right)}^{3}+{\text{b}}_{m}{\left(\alpha -\alpha_{m}\right)}^{2}+c_{m}\left(\alpha -\alpha_{m}\right)+d_{m}, & \alpha \in [\alpha_{m},\alpha_{m+1}) \end{array}$$

where **a***_m_*, **b***_m_*, **c***_m_* and **d***_m_* are the piecewise polynomial coefficient row vectors obtained by applying cubic spline interpolation in the tensor coefficient space between the closest two adjacent training target types **i**(α*_m_*) and **i**(α*_m_*_+1_), as depicted in Figure 5. Let Ψ(**Θ**) be the RBF mapping along the view manifold given by:

(9) $$\Psi \left(\Theta \right)=\left[\kappa \left(\left\|\Theta -{\text{S}}_{1}\right\|\right),\ldots ,\kappa \left(\left\|\Theta -{\text{S}}_{N_{c}}\right\|\right)\right]$$

where *κ*(‖**Θ** − **S***_i_*‖) = *e*^−^*^c^*^(^**^Θ^**^−^**^S^***^_i_^*^)^*^^T^^* ^(^**^Θ^**^−^**^S^***^_i_^*^)^, *c* is the RBF kernel width, **S***_i_* is a training view on the view manifold and *N_c_* is the number of training views. One major advantage of this DCT-based shape representation over the original silhouette-based one is that it naturally provides reconstruction of a shape at arbitrary magnification factors by appropriately zero-padding the DCT coefficients prior to inverse DCT (IDCT). This feature is desirable to deal with various IR targets at different ranges.

We represent the shape embedding function **Φ** (referred to in Figure 3c and Equation (3)) in terms of the CVIM parameter **Λ** by:

(10) $$\Phi \left(\Lambda \right)=\text{IDCT}\left({\text{S}}_{\text{DCT}}\left(\Lambda \right)\right)$$

where IDCT(·) is the IDCT with two reshape operations. The first is for the input (from 1D to 2D) prior to IDCT, and the second is for the output (from 2D to 1D) after IDCT. Note that the derivative of the IDCT of a matrix may be computed as the IDCT of the derivative of that matrix [36]. Therefore, the DCT-shape presentation can easily be incorporated into the above optimization framework without major modifications. Through this CVIM model, target shapes corresponding to arbitrary α can readily be interpolated along the view and identity manifolds.

**Figure 6:**
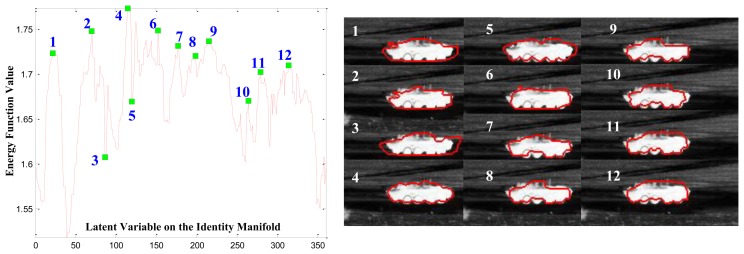
Effectiveness of the energy function. (Left) Plot of the energy function with respect to the identity latent variable; (right) a group of CVIM-generated shapes corresponding to the latent variables labeled in the plot on the left are superimposed onto the original IR data chips.

Optimizing the posterior Equation (2) may be thought of as finding a contour that maximizes the histogram difference between the foreground and background in the region of interest. This consideration is based on the assumption that the target-of-interest has different intensity values compared with the background. Intuitively, if the shape contour of the target is correctly hypothesized in terms of the target type (recognition), view angle (pose estimation) and location (segmentation), then the foreground and background defined by this contour will have maximum histogram divergence and, therefore, maximize the energy function Equation (2) as illustrated in Figure 6. For a given observation, in Figure 6, we calculate the value of the energy function with respect to almost all possible values along the circularly-shaped identity manifold α = 1, 2, 3, …, 360° with the view angle **Θ** known for simplicity. The figure shows several CVIM interpolated shapes superimposed on the original mid-wave IR image data. As seen in the left part of the figure, the maximum value of the energy function is attained by the contour (numbered 4) that is best in the sense of being closest to the actual boundary of the target in the right part of the figure. However, the multi-model nature of the energy function as shown in Figure 6 (left part), which is typical, represents significant challenges for CVIM-based shape optimization and motivates the PSO and GB-PSO algorithms that we develop below in Sections 3.3 and 3.4.

### 3.3. PSO-Based Optimization

We first implement a standard PSO algorithm due to its simplicity and effectiveness in dealing with multi-modal optimization problems. PSO algorithms were originally developed in [38] inspired by the observation of bird flocking and fish schooling, which are also related to genetic algorithms, and they are widely used in both scientific research [66,67] and engineering applications [68,69]. PSO optimizes a problem by moving solution hypotheses around in the search-space according to the current hypothesis and velocity computed to the present local and global optima. Our energy function is defined in Equation (2). Since we assume **Λ** = [θ, ϕ, α]*^T^* (CVIM parameters) to be uniformly distributed (*i.e.*, no prior knowledge) with the registration of the object frame **p** omitted, the energy function Equation (2) is rewritten as:

(11) $$f\left(\Lambda \right)=P\left(\Lambda |\Omega \right)\propto \prod_{i=1}^{N}\left\{H_{\epsilon }\left(\Phi_{{\text{x}}_{i}}\left(\Lambda \right)\right)P_{f}+\left(1-H_{\epsilon }\left(\Phi_{{\text{x}}_{i}}\left(\Lambda \right)\right)\right)P_{b}\right\}$$

where **Φ**_x*_i_*_ (**Λ**) is defined as the value of shape embedding (in the form of the signed distance transform) in Equation (10) at pixel location **x***_i_*. During the PSO optimization process, particles are updated as flying in the latent space of CVIM, **Λ**, based on the velocity **V**:

(12) $$\Lambda_{j}\left(k+1\right)=\Lambda_{j}\left(k\right)+{\text{V}}_{j}\left(k+1\right)$$

where *j* and *k* are the particle and iteration indexes, respectively. Velocity **V** is a randomly-weighted average of the best position evaluated by that particle so far and the global best position among all particles:

(13) $${\text{V}}_{j}\left(k+1\right)={\text{V}}_{j}\left(k\right)+{ϒ}_{1}\cdot ({\text{L}}_{j}^{\text{best}}\left(k\right)-\Lambda_{j}\left(k\right)+{ϒ}_{2}\cdot \left(G^{\text{best}}\left(k\right)-\Lambda_{j}\left(k\right)\right)$$

where **V***_j_*(*k*) is the velocity for particle *j* = 1 : *ps* at optimization step *k* = 1 : *K* and *ps* is the population size. **ϒ**_1_ and **ϒ**_2_ are random vectors, where each entry is uniformly distributed between [0,1]. ${\text{L}}_{j}^{\text{best}}\left(k\right)$ is the best position in the latent space found by particle *j* evaluated by Equation (11), while **G***^best^*(*k*) is the global best position found among all particles. It is worth mentioning that the direction of each particle move is determined by comparing the current energy with the present local/global optima. Thus, while the magnitude of the move is chosen randomly, the direction is not. By doing so, PSO discourages the solution from becoming trapped in local optima by moving each particle in a way that considers both the local and global best solutions from among all current particles. All particle hypotheses are clipped to be within the range of the CVIM latent space, and the maximum velocity is restricted within ±10% of the range of the latent space [70]. We summarize the PSO algorithm in Algorithm 1.

### 3.4. GB-PSO-Based Optimization

The PSO algorithm is simple, straightforward and robust, but it suffers high computational load due to CVIM-based shape interpolation, as well as the large number of iterations that are typically needed to obtain convergence. In some applications, the gradient is incorporated in sampling optimization to achieve a higher convergence rate [71]. In this section, we take advantage of the parametric nature of CVIM and incorporate a gradient-ascent step in the PSO to obtain a gradient-boosted PSO (GB-PSO) that overcomes these limitations by balancing between exploration and convergence with a deterministic and fast local search. Thus, GB-PSO is expected to be both more efficient and effective than the basic PSO in Algorithm 1.



**Algorithm 1** PSO method.
1:**Initialization**2:• do level-set segmentation to initialize the target location **p**3:• draw particles **Λ***_j_*(0), *j* = 1 : *ps* randomly distributed in the latent space **Λ**, where *ps* is the population size4:• evaluate **Λ***_j_*(0) by Equation (11) to get *f*(**Λ***_j_*(0)), set **G***^best^*(0) = arg max**_**Λ**_***_j_*(0) *f*(**Λ***_j_*(0)) and 
${\text{L}}_{j}^{\text{best}}\left(0\right)=\Lambda_{j}\left(0\right)$, (*j* = 1 : *ps*)5:**PSO algorithm**6:**for** each iteration (*k* = 0 : *K* − 1) **do**7: **for** each particle (*j* = 1 : ps) **do**8:  • calculate velocity **V***_j_*(*k* + 1) and new particle **Λ***_j_*(*k* + 1) by Equations (13) and (12)9:  • compute *f*(**Λ***_j_*(*k* + 1)) by Equation (11)10:  **if**
$f\left(\Lambda_{j}\left(k+1\right)\right)>f\left({\text{L}}_{j}^{\text{best}}\left(k\right)\right)$
**then**11:   • set 
${\text{L}}_{j}^{\text{best}}\left(k+1\right)=\Lambda_{j}\left(k+1\right))$12:   **if**
*f*(**Λ***_j_*(*k* + 1)) > *f*(**G***^best^*(*k*)) **then**13:    • set **G***^best^*(*k* + 1) = **Λ***_j_*(*k* + 1))14:   **end if**15:  **end if**16: **end for**17:**end for**18:• obtain the final result, Λ*, by selecting from **G***^best^*(*K*).


A classical gradient ascent method starts from an initial hypothesis in the search space, *i.e.*, the parameter space of CVIM denoted by **Λ**; then, by computing the local gradient direction, small steps are made toward the maximum iteratively. Due to the smooth and continuous nature of CVIM, which generates the shape embedding function **Φ**, *f* = *P*(**Λ**| Ω) can be differentiated with respect to **Λ**. Beginning from some initial guesses **Λ**_0_, we will then update our guess iteratively along the gradient direction:

(14) $$\Lambda^{\left(k+1\right)}=\Lambda^{\left(k\right)}-r\cdot \left(-\nabla f{|}_{\Lambda =\Lambda^{\left(k\right)}}\right),=\Lambda_{k}+r\cdot \nabla f{|}_{\Lambda =\Lambda^{\left(k\right)}}$$

where *r* is the learning rate that determines the step size and ∇*f*|**_Λ_**_=_**_Λ_***_k_* is the gradient of *f* evaluated at the old guess. To compute ∇*f*|**_Λ_**_=_**_Λ_***_k_*, we take the derivative of *f* with respect to **Λ** by the chain rule as:

(15) $$\frac{\partial f}{\partial \Lambda }={\left({\left(\frac{\partial f}{\partial \Phi }\right)}^{T}\cdot \frac{\partial \Phi }{\partial \Lambda }\right)}^{T}={\left(\frac{\partial \Phi }{\partial \Lambda }\right)}^{T}\frac{\partial f}{\partial \Phi }$$

Similar to [61], the first term in Equation (15) can be written as:

(16) $$\frac{\partial f}{\partial \Phi }=\frac{\delta_{\epsilon }\left(\Phi \right)\left(P_{f}-P_{b}\right)}{H_{\epsilon }\left(\Phi \right)P_{f}+\left(1-H_{\epsilon }\left(\Phi \right)\right)P_{b}}$$

where δ_∊_(·) is the derivative of the Heaviside step function and *P_f_* and *P_b_* are defined in Equations (4) and (5). Since the latent variable **Λ** = [**Θ***^T^*, α]*^T^*, so the second term in Equation (15) may be written as:

(17) $$\frac{\partial \Phi }{\partial \Lambda }={\left[\begin{array}{l} \text{IDCT}{\left(\frac{\partial S_{\text{DCT}}}{\partial \Theta }\right)}^{T}\\ \text{IDCT}{\left(\frac{\partial S_{\text{DCT}}}{\partial \alpha }\right)}^{T} \end{array}\right]}^{T}$$

The CVIM-based DCT generation of 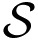
_DCT_ is defined in Equation (7). From the properties of the tensor multiplication [72], we can rewrite Equation (7) as:

(18) $$S_{\text{DCT}}\left(\alpha ,\Theta \right)=\text{A}\times_{3}\text{i}\left(\alpha \right)\times_{2}\Psi \left(\Theta \right)=\text{A}\times_{2}\Psi \left(\Theta \right)\times_{3}\text{i}\left(\alpha \right)$$

where both **i**(α) and Ψ(**Θ**) are row vectors. The steepest ascent optimization may then be performed based on the gradients along the view and identity manifolds.

(I) **Gradient along the view manifold**
  Let **B** = **A** ×_3_ **i**(α). From the tensor multiplication and flattening properties [72], it then follows that:
  (19) $$S_{\text{DCT}}\left(\alpha ,\Theta \right)=\text{B}\times_{2}\Psi \left(\Theta \right)={\text{B}}_{\left(2\right)}^{T}\cdot \Psi^{T}\left(\Theta \right)$$
  where **B**_(2)_ is the mode-two flattened matrix of **B**. Hence,
  (20) $$\frac{\partial S_{\text{DCT}}}{\partial \Theta }={\text{B}}_{\left(2\right)}^{T}\cdot \frac{\partial \Psi^{T}\left(\Theta \right)}{\partial \Theta }$$
  It then follows from Equation (9) that:
  (21) $$\frac{\partial \Psi^{T}\left(\Theta \right)}{\partial \Theta }=-2c{\left[\kappa \left(\left\|\Theta -{\text{S}}_{1}\right\|\right)\left(\Theta -{\text{S}}_{1}\right),\ldots ,\kappa \left(\left\|\Theta -{\text{S}}_{N_{c}}\right\|\right)\left(\Theta -{\text{S}}_{N_{c}}\right)\right]}^{T}$$
  where *κ*(‖**Θ** − **S***_i_*‖) is defined in Equation (9). For the first term in Equation (17), we then have:
  (22) $$\frac{\partial S_{\text{DCT}}}{\partial \Theta }=-2c\cdot {\text{B}}_{\left(2\right)}^{T}\cdot {\left[\kappa \left(\left\|\Theta -{\text{S}}_{1}\right\|\right)\left(\Theta -{\text{S}}_{1}\right),\ldots ,\kappa \left(\left\|\Theta -{\text{S}}_{N_{c}}\right\|\right)\left(\Theta -{\text{S}}_{N_{c}}\right)\right]}^{T}$$
(II) **Gradient along the identity manifold**
  Let **C** = **A** ×_2_ Ψ(**Θ**). From Equation (18), we have then that:
  (23) $$S_{\text{DCT}}\left(\alpha ,\Theta \right)=\text{C}\times_{3}\text{i}\left(\alpha \right)={\text{C}}_{\left(3\right)}^{T}\cdot {\text{i}}^{T}\left(\alpha \right)$$
  so:
  (24) $$\frac{\partial S_{\text{DCT}}}{\partial \alpha }={\text{C}}_{\left(3\right)}^{T}\cdot \frac{\partial {\text{i}}^{T}\left(\alpha \right)}{\partial \alpha }$$
  Since **i**(α) is the piecewise polynomial interpolation function, which is differentiable between any two given data points, it follows from Equation (8) that:
  (25) $$\begin{array}{cc} \frac{\partial {\text{i}}^{T}\left(\alpha \right)}{\partial \alpha }=3{\text{a}}_{m}^{T}{\left(\alpha -\alpha_{m}\right)}^{2}+2{\text{b}}_{m}^{T}\left(\alpha -\alpha_{m}\right)+{\text{c}}_{m}^{T}, & \alpha \in [\alpha_{m},\alpha_{m+1}) \end{array}$$
  Thus, we obtain finally:
  (26) $$\begin{array}{cc} \frac{\partial S_{\text{DCT}}}{\partial \alpha }={\text{C}}_{\left(3\right)}^{T}\cdot \left[3{\text{a}}_{m}^{T}{\left(\alpha -\alpha_{m}\right)}^{2}+2{\text{b}}_{m}^{T}\left(\alpha -\alpha_{m}\right)+{\text{c}}_{m}^{T}\right], & \alpha \in [\alpha_{m},\alpha_{m+1}) \end{array}$$
  which together with Equation (22) provides the explicit formulation for both terms in Equation (17)
(III) **Gradient in the latent space**
  From Equations (16) and (17), ∇*f*|**_Λ_**_=_**_Λ_***_k_* may be rewritten as:
  (27) $$\nabla f{|}_{\Lambda =\Lambda_{k}}={\left[\begin{array}{l} \text{IDCT}{\left(\frac{\partial S_{\text{DCT}}}{\partial \Theta_{k}}\right)}^{T}\\ \text{IDCT}{\left(\frac{\partial S_{\text{DCT}}}{\partial \alpha_{k}}\right)}^{T} \end{array}\right]}^{T}\frac{\delta_{\in }\left(\Phi_{k}\right)\left(P_{f}-P_{b}\right)}{H_{\epsilon }\left(\Phi_{k}\right)P_{f}+\left(1-H_{\in }\left(\Phi_{k}\right)\right)P_{b}}$$
  where α ∈ [α*_m_*, α*_m_*_+1_), **Φ***_k_* = **Φ**(**Λ***_k_*) and *P*(**x**| **Λ***_k_*, **y**) are defined in Equations (3) and (10), while $\frac{\partial S_{\text{DCT}}}{\partial \Theta_{k}}$ and $\frac{\partial S_{\text{DCT}}}{\partial \alpha_{k}}$ are defined in Equations (22) and (26).

**Algorithm 2** The gradient-boosted (GB)-PSO method.
1:**Initialization**2:• refer to Algorithm 1 Lines 1 ∼ 43:**GB-PSO step**4:**for** each iteration step (*k* = 0 : *K* − 1) **do**5: **for** each particle (*j* = 1 : *ps*) **do**6:  • refer to Algorithm 1 Lines 8 ∼ 157: **end for**8: **Gradient Ascent Local Search**9: • set **Λ**^0^ = **G***^best^*(*k* + 1) for gradient ascent local search;10: **for** each gradient ascent step (*l* = 1 : *pl*) **do**11:  • calculate ∇*f*|**_Λ_**_=_**_Λ_***_l_*_−1_12:  **if** the gradient is not significant **then**13:   break14:  **end if**15:  • draw a sample for step size *r*16:  • update **Λ***^l^* = **Λ***^l^*^−1^ + *r* · ∇*f*|**_Λ_**_=_**_Λ_**_−1_ according to Equation (27)17: **end for**18: • set **G**^best^(*k* + 1) = **Λ***^l^*;19:**end for**20:• obtain the final result, Λ*, by selecting from **G**^best^(*K*).


As suggested in [73], a uniformly-distributed random step size (*r*) could be used for the steepest ascent method, which turned out to be effective in practice. In practice, *r* is uniformly distributed between [ $\frac{\pi }{90}$, $\frac{\pi }{15}$]. In the GB-PSO method, the standard PSO is involved as the first step, then the global optimum (**G***^best^*(*k* + 1)) is updated by the gradient ascent method, which helps the next round PSO converge fast by improving velocity estimation. Thus, in the GB-PSO method, the total number of iterations required could be dramatically reduced compared with PSO. The computational load of the additional steps in GB-PSO is negligible due to two reasons: (1) the analytical nature of the energy function makes the gradient computation very efficient for the present global solution **G***^best^*(*k* + 1) that is to be shared by all particles in the next iteration; (2) the update along the gradient direction is done analytically according to Equation (27), and there is a maximum number of gradient ascent iterations (*i.e.*, *pl* = 20 in this work) and a check of the current gradient value to determine if additional moves are necessary. In our experiment, we found that the actual number of the steps along the gradient direction is often much less than *pl* (around 10), which confirms that the solution of gradient-based search is in the proximity of a local optimum. We summarize the GB-PSO method in Algorithm 2.

## 4. Experimental Result

In this work, our interest is to develop a general model-driven ATR algorithm where no IR data are used for training and no prior feature extraction is needed from the IR data, unlike most traditional methods that heavily rely on the quality of the training data, as well as feature extraction. We have conducted two comparative studies to evaluate the performance of the proposed algorithms. First, we have involved five comparable algorithms, including the proposed PSO and GB-PSO algorithms, all of which apply CVIM for shape modeling. Second, we also compared our algorithms with several recent ATR algorithms, including two SRC-based approaches [45,46] and our previously-proposed ATR algorithm, which involves a joint view-identity manifold (JVIM) for target tracking and recognition [37]. The purpose of the first study is to validate the advantages of “implicit shape matching” over “explicit shape matching”, as well as the efficiency of GB-PSO over PSO. That of the second study is to demonstrate the effectiveness of our new ATR algorithms compared with the recent ones in a similar experimental setting. It was reported in [14] that SRC-based methods can achieve state-of-the-art performance. In the following, we will first discuss the experimental setup shared by two comparative studies along with the metrics used for performance evaluation. Then, we present the two comparative studies one-by-one in detail.

### 4.1. Experimental Setup

**Figure 7:**
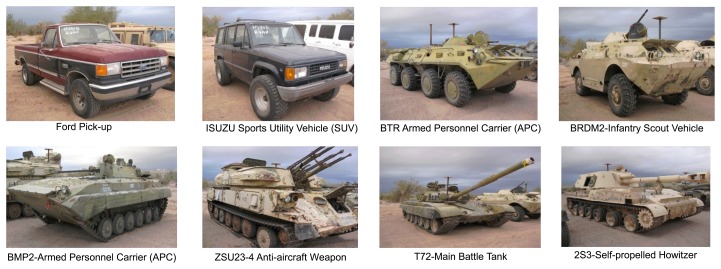
All eight targets we used for algorithm evaluation were from the SENSIAC database.

Similar to [33], we selected six 3D CAD models for each of the six target classes for CVIM training (36 models in total; Figure 4): APCs (armored personnel carriers), tanks, pick-ups, sedans, vans and SUVs. We considered elevation angles in 0°∼40° and azimuth angles in 0°∼360°, with 10° and 12° intervals along the elevation and azimuth angles, respectively, on the view manifold, giving 150 multi-view shapes for each target. We also adopted a DCT-based shape descriptor [36], which facilitates CVIM learning and shape inference. All experiments were performed against the SENSIAC database [5], which provides a large collection of mid-wave IR and visible data depicting seven military targets and two civilian vehicles. We used 24 mid-wave (23 night-time and 1 day-time) IR sequences captured from 8 targets (Figure 7) at 1 km, 2 km and 3 km. In each sequence, there is a civilian or military vehicle traversing a closed-circular path with a diameter of 100 m. We selected 100 frames from each sequence by down-sampling each sequence that has 1800 frames originally, where the aspect angle ranges from 0° to 360° with around a 5°–10° interval; so in total, there are 2400 frames used for evaluation. The SENSIAC database also provides a rich amount of metadata, which can be used for performance evaluation, such as the aspect angle of the target, the field of view and the 2*D* bounding box of the target in each frame. Since we mainly focus on the recognition rather than detection, we also generated our target chips with the help of target 2D locations from this metadata (averaging around 50 × 30 = 1500 pixels at 1 km, 25 × 14 = 350 pixels at 2 km and 15 × 10 = 150 pixels at 3 km) in our experiments.

### 4.2. The First Comparative Study: CVIM-Based Approaches

This study compares five CVIM-based algorithms, which involve different segmentation and optimization techniques. Specifically, Method I uses background subtraction [74] to pre-segment a target-of-interest. This method is only suitable for a stationary sensor platform. Method II applies level set segmentation without a shape prior [61]. Both Method I and Method II need explicit shape matching, which involves Markov Chain Monte Carlo (MCMC)-based CVIM inference after segmentation to accomplish ATR [75]. Method III applies a multi-threaded MCMC-based inference technique to jointly optimize over CVIM in a level set by involving implicit shape matching without target pre-segmentation. It was shown in [75] that Method III significantly outperforms the first two, but it suffers from high computational complexity due to the MCMC-based shape inference. PSO and GB-PSO are referred to as Methods IV and V, respectively. The computational time for each ATR chip (50 × 30 pixels) for five algorithms is around 10, 14, 22, 15 and 6 s, respectively, using an un-optimized MATLAB code on a PC with a Quad-core CPU (2.5 GHZ).

We evaluate these five algorithms with respect to: (1) the accuracy of pose estimation (*i.e.*, the aspect angle); (2) the 2D pixel location errors between the segmented shape and the ground truth bounding box; (3) the recognition accuracy in terms of six major target classes; and (4) the sensor-target distance (*i.e.*, range, computed by scaling factors) errors in meters. To examine the robustness of our algorithms, we analyze (5) the recognition accuracy *versus* three related factors, *i.e.*, the contrast of image chips [76], the foreground/background χ^2^ histogram distance [77] based on the segmentation results and the aspect angle. The chip contrast and χ^2^ histogram distance indicate the IR image quality and the target visibility, respectively. Similar to [78–81], we also evaluate the overlap ratio between the estimated bounding box (derived from the segmentation result) and the ground truth bounding box (available from ground-truth data) (6), which is a simple, yet effective and widely-accepted way to quantify the segmentation performance. Furthermore, we manually created the ground truth segmentation masks from five randomly-selected frames per IR sequence, so that we can compute the overlap ratio between the segmentation results with the ground-truth masks (7). Moreover we will show the capability of the proposed algorithm (GB-PSO) for sub-class recognition, *i.e.*, the specific target type within a class, even if the exact target type is not in the training data.

#### 4.2.1. Pose Estimation Results

**Figure 8:**
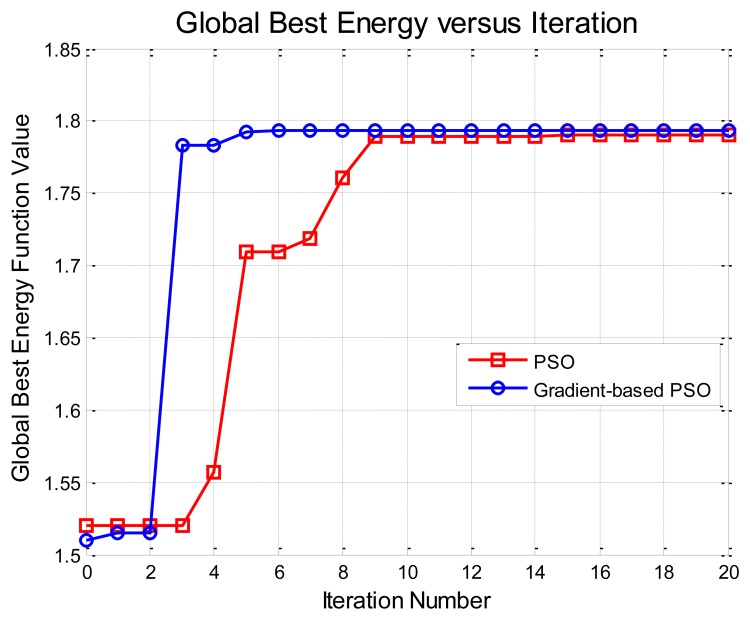
Convergence plots for PSO and GB-PSO in one example.

Table 1 reports aspect angle error (pose estimation) results for all five tested methods along with the 2D pixel error and 3D range error in the predefined 3D camera coordinate system (given in the metadata). We can see clearly that both Methods IV and V can achieve moderate, significant and slight improvements over Method I (background subtraction for segmentation), Method II (level set segmentation without shape prior) and Method III (MCMC-based CVIM inference), respectively. Although Methods IV and V do not provide a significant improvement in pose and location estimation performance compared to Method III, they provide similar performance at a greatly reduced computational complexity. Numerical results from PSO and GB-PSO are comparable to each other. However, Figure 8 shows that GB-PSO converges nearly three-times faster than the PSO, demonstrating the value of gradient boosting in the CVIM latent space.

#### 4.2.2. Target Recognition Results

**Figure 9:**
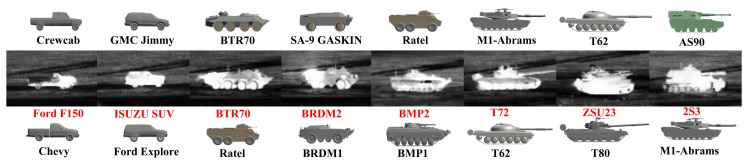
Some sub-class recognition results under 1000 m. The first row and third row show the closest training vehicles along the identity manifold in the CVIM, and the middle row presents the original IR chips.

The recognition results are computed based on the percentage of frames where the target class is correctly classified. As shown in Table 2, both PSO (Method IV) and GB-PSO (Method V) generally achieve modest performance gains over Method I–III, while GB-PSO does so with a significantly reduced computational complexity compared to all four of the other methods. Furthermore, Figure 9 shows some sub-class recognition results for eight 1-km IR images. The sub-class recognition can be achieved via CVIM by finding the two closest training target types along the identity manifold. Since the training data only have the BTR70 model, we find that we can recognize the BTR70 at the sub-class level most of the time. Interestingly, we can see that T72, BMP2 and 2S3 are also recognized as T62, BMP1 and AS90, respectively, which are the closest sub-class target types available in our training data.

**Figure 10:**
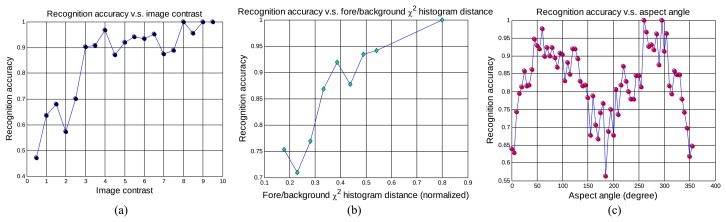
Robustness analysis of GB-PSO over all 2400 chips. (**a**) Recognition accuracy versus the chip contrast; (**b**) recognition accuracy *versus* foreground/background histogram distances; (**c**) recognition accuracy *versus* the aspect angles.

We also summarize recognition results from GB-PSO vs. the chip contrast, foreground/background histogram distance and aspect angle in Figure 10. It is shown in Figure 10a that our algorithm performs well for most chips with reasonable contrast and tends to deteriorate for chips with a very low contrast, which is usually associated with poor image quality (e.g., day-time IR imagery). As illustrated in Figure 10b, the foreground/background χ^2^ histogram distance is strongly related to the recognition accuracy. This is because the χ^2^ distance is related to the target visibility and can also quantify the segmentation quality. When segmentation results are good with large background/foreground separation (large χ^2^ distance values), the recognition accuracies are usually high, which also imply good target segmentations. Furthermore, the aspect angle is a key factor that affects the recognition performance. As shown in Figure 10c, the highest accuracy occurs around the side views (90° and 270°) when the targets are most recognizable. Most failed cases are around 0° (or 360°) (frontal views) and 180° (rear views), when it is hard to differentiate different targets due to the high shape ambiguity. Since we only use the shape information here, a more advanced and informative target appearance representation that involves intensity and other features could make ATR performance more robust to aspect angles.

#### 4.2.3. Target Segmentation Results

**Figure 11:**
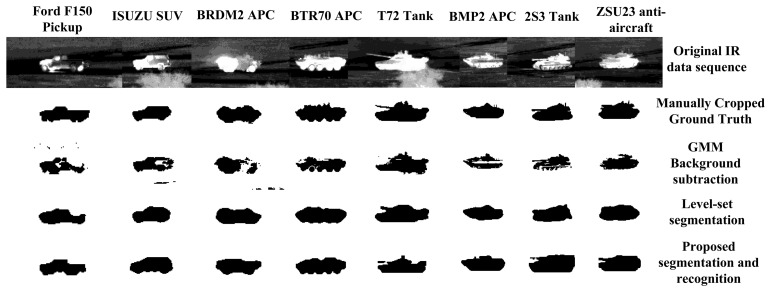
Snapshot of the segmentation results. From the first row to the last: original IR frame, manually-cropped segmentation masks, results of background subtraction segmentation, level set segmentation without shape prior and the final segmentation and recognition results with CVIM shape prior (Method IV) interpolated from the CVIM.

Table 2 shows the target segmentation results in terms of the bounding box overlap, the segmentation mask overlap and the foreground/background χ^2^ histogram distance. Both PSO and GB-PSO outperform Methods I and II, while performing comparably to Method III at a lower computational complexity. Figure 11 shows some snapshots of the original IR imagery of eight targets under the 1-km range, along with the manually-cropped segmentation masks, the results of the background subtraction segmentation, the level set segmentation without a shape prior and the PSO method, respectively. It may be seen that the CVIM shape prior drives the segmentation to a semantically more meaningful shape compared to Methods I and II, where a shape prior is not involved.

**Figure 12:**
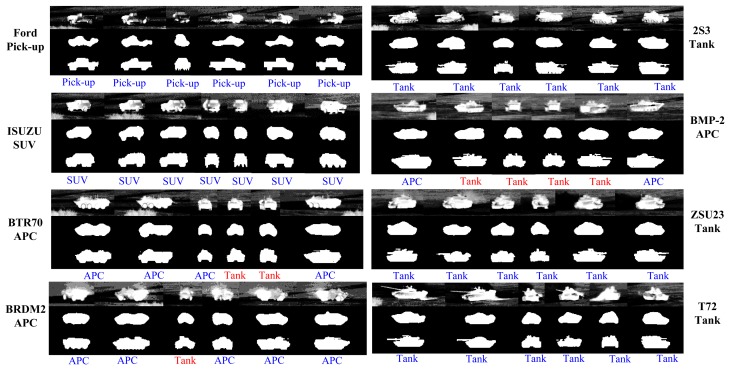
Some GB-PSO results for pose estimation and recognition results of eight targets under a 1-km target-sensor range from the SENSIAC database. For each target, from the first row to the third: original IR frame, level set segmentation-based initialization and the final pose estimation and recognition results from GB-PSO. Recognition results are denoted in blue if correct and red if wrong below each chip.

Some snapshots of segmentation results along with pose estimation and recognition results of Method V (GB-PSO) are shown in Figure 12. It is found that GB-PSO is robust to background clutter and engine smoke, and the frontal/rear views may pose some challenge. For BRDM2 APC, we see a heat spot near the tail, which changes the target appearance significantly, but we can still recognize it as an APC. However, for targets of near front/rear views, although the segmentation results are still acceptable, the recognition results are often wrong. For example, the BMP2 APC was misrecognized as the M60 tank in the front view. By closely observing the IR appearances of BMP2 APC, we find that this particular APC does indeed look similar to a tank when viewed frontally. This can also be explained by our identity manifold topology learned by *class-constrained shortest-closed-path*, where the BMP2 stays closest to tanks along the identity manifold among all APCs, as shown in Figure 4.

**Figure 13:**
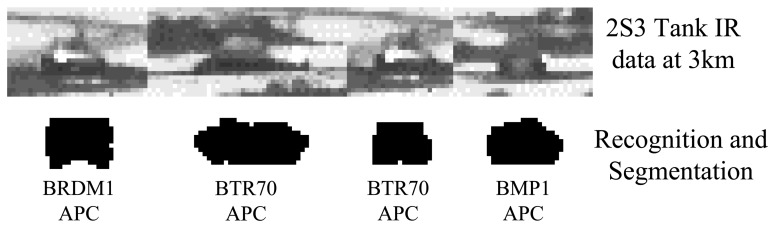
Some failed cases of the 2S3 tank in a day-time sequence at 3 km.

A similar case happens to BTR70 APC. Moreover, the proposed algorithm as realized in Methods IV and V performs poorly against long-range day-time IR data (3 km), where the foreground/background contrast is low and the target is small. This is illustrated in Figure 13, where the 2S3 tank is misclassified as an APC in several frames. As we already mentioned, a more powerful appearance representation is needed to handle challenging cases of this type.

### 4.3. Comparative Study: Recent ATR Methods

This comparative study includes three recent ATR algorithms that are compared against PSO and GB-PSO. Specifically, we applied the gradient-based optimization technique discussed in [37] to apply JVIM (learned from the same set of training shapes as CVIM) for image-based ATR where level set segmentation without a shape prior is used for initialization. In addition, we have also implemented the SRC-based algorithm [14] and the multi-attribute Lasso with group constraint (MALGC) [46], which is an extended SRC approach by taking advantage of the attribute information (*i.e.*, angles) during sparse optimization. Both SRC algorithms require a dictionary that includes training shapes also used for CVIM learning. The input is the level set segmentation output from an IR chip. We also use the segmentation results from GB-PSO for SRC-based ATR (namely SRC-GB-PSO) to see the effect of good target segmentation. The computational time (in the same computational setting as before) for four implementations is around 4 (JVIM), 16 (SRC), 20 (MALGC) and 18 (SRC-GB-PSO) s, compared with 15 and 6 s for PSO and GB-PSO, respectively. We compare six ATR algorithms in Table 3.

We have the following observations and discussion according to Table 3.

- The JVIM model unifies the view and identity manifolds in one latent space for more accurate shape modeling than CVIM, which involves separate view and identity manifolds [82], and it is especially suitable for target tracking due to the unified and smooth shape manifold [37]. However, JVIM has a similar multi-modal problem as CVIM that makes the gradient-based optimization often trapped in local minima, as reflected by relatively poor results in Table 3. It may not be efficient to apply sample-based approaches (e.g., PSO or MCMC) to JVIM optimization due to the fact that its joint manifold structure will require a large number of samples to ensure effective sampling. The reason that JVIM shows promising results in target tracking is because the dynamic modeling involved greatly facilitates sequential state estimation. In the case of image-based ATR, CVIM shows some advantages over JVIM due to its simpler structure.
- Both SRC and MALGC methods show reasonable performance by only using shapes segmented by level set for ATR. Especially for the range of 1 km when target segmentation is likely more accurate, all algorithms are very comparable. It is observed that MALGC is slightly better than SRC by utilizing the angle information in sparse optimization. More interestingly, we can see that better target segmentation results via GB-PSO moderately improve the SRC's performance (the fourth algorithm, GB-PSO-SRC). The computational complexity of SRC and MALGC is comparable with PSO and much higher than that of GB-PSO.
- It is shown that the proposed PSO and GB-PSO algorithms are comparable, both of which are better than the others in all cases. Although the improvement of GB-PSO over the other two SRC approaches is moderate, it does offer several advantages: (1) it is computationally efficient with only a 30%–40% computational load; (2) target segmentation is not required and can be considered as a byproduct of ATR; and (3) the proposed GB-PSO algorithm is a model-driven approach that does not require real-world training data, and it has potential to be combined with other data-driven approaches to further improve ATR performance.

## 5. Conclusions

In this paper, we have integrated a shape generative model (CVIM) into a probabilistic level set framework to implement joint target recognition, segmentation and pose estimation in IR imagery. Due to the multi-modal nature of the optimization problem, we first implemented a PSO-based method to jointly optimize CVIM-based implicit shape matching and level set segmentation and then developed the gradient-boosted PSO (GB-PSO) algorithm to further improve the efficiency by taking advantage of the analytical and differentiable nature of CVIM. We have conducted two comparative studies on the recent SENSIAC ATR database to demonstrate the advantages of the two PSO-based algorithms. The first study involves five methods where CVIM is optimized by either explicit shape matching or MCMC-based implicit shape matching. GB-PSO and PSO are shown to be more effective than other methods. Moreover, GB-PSO was also shown to be more efficient than PSO with a much improved convergence rate due to the gradient-driven technique. The second study includes a few recent ATR algorithms for performance evaluation. It is shown that the proposed GB-PSO algorithm moderately outperforms other recent ATR algorithms at a much lower computational load. The proposed framework could be further extended to incorporate new appearance features or effective optimization to deal with more challenging ATR problems.

## Figures and Tables

**Table 1:** Pose and location estimation errors for all five tested methods averaged over SENSIAC mid-wave IR sequences for each target to sensor distances depicting eight difference targets (Method I/Method II/Method III/Method IV/Method V).

	**2D Pixel Error (pixels)**	**Aspect Angle Error (°)**	**Range Error (m)**
1 km	2.8/3.1/1.9/2.1/**1.9**	17.2/17.9/15.1/**14.8**/15.1	25.1/27.8/24.2/**24.1**/24.5
2 km	2.9/3.4/2.3/2.4/**2.1**	21.2/25.2/18.7/18.2/**17.1**	39.1/38.2/33.8/32.8/**32.6**
3 km	2.5/3.8/2.2/**1.8**/2.0	26.1/27.5/21.7/21.9/**20.5**	43.5/48.3/40.2/**40.1**/41.1

**Table 2:** Overall recognition and segmentation results of five methods (I/II/III/IV/V).

	**Average Recognition Accuracy (%)**	**Bounding Box Overlap (%)**

1 km	81/78/85/**86**/85	85.2/82.9/88.1/88.6/**88.9**
2 km	71/64/73/75/**76**	75.6/74.1/79.5/**79.8**/79.2
3 km	69/62/70/72/**73**	67.7/65.5/70.1/70.9/**71.6**

	**Segmentation Mask Overlap (%)**	**Fore/Background χ^2^ Histogram Distance**

1 km	79.3/83.2/83.5/**83.8**/83.6	0.30/0.32/0.34/0.34/**0.35**
2 km	72.3/73.9/79.8/79.6/**80.1**	0.26/0.25/0.29/0.28/**0.31**
3 km	63.8/67.2/68.8/**69.3**/69.1	0.20/0.23/0.25/**0.26**/0.25

**Table 3:** The performance comparison with recent ATR methods in terms of the recognition accuracy and the aspect angle error (joint view-identity manifold (JVIM)/sparse representation-based classification (SRC)/multi-attribute Lasso with group constraint (MALGC)/SRC-GB-PSO/PSO/GB-PSO).

**Ranges**	**Average Recognition Accuracy (%)**	**Aspect Angle Error (°)**
1 km	82/83/83/85/**86**/85	16.9/15.6/15.4/15.3/**14.8**/15.1
2 km	69/72/73/75/75/**76**	22.3/20.1/19.9/17.8/18.2/**17.1**
3 km	65/69/70/72/72/**73**	26.8/24.5/23.9/21.1/21.9/**20.5**
